# Oxidative stress and male infertility

**DOI:** 10.1002/rmb2.12353

**Published:** 2020-10-18

**Authors:** Teppei Takeshima, Kimitsugu Usui, Kohei Mori, Takuo Asai, Kengo Yasuda, Shinnosuke Kuroda, Yasushi Yumura

**Affiliations:** ^1^ Department of Urology, Reproduction Center Yokohama City University Medical Center Yokohama city Japan

**Keywords:** antioxidants, lipid peroxidation, male infertility, oxidative stress, reactive oxygen species

## Abstract

**Background:**

Between 30% and 80% of patients with male infertility produce excessive reactive oxygen species (ROS) in their ejaculate even though the cause of male infertility is unexplained in approximately half of cases. The strong connection between oxidative stress (OS) and male infertility has led recent investigators to propose the term “Male Oxidative Stress Infertility (MOSI)” to describe OS‐associated male infertility.

**Methods:**

We searched the PubMed database for original and review articles to survey the effects of OS on male infertility, and then verified the effects and treatments.

**Main findings:**

Seminal plasma contains many antioxidants that protect sperm from ROS, because low amounts of ROS are required in the physiological fertilization process. The production of excessive ROS causes OS which can lower fertility through lipid peroxidation, sperm DNA damage, and apoptosis. Several assays are available for evaluating OS, including the MiOXSYS® analyzer to measure oxidation‐reduction potential. Several measures should be considered for minimizing OS and improving clinical outcomes.

**Conclusion:**

Accurately diagnosing patients with MOSI and identifying highly sensitive biomarkers through proteomics technology is vital for better clinical outcomes.

## Graphical abstract

Excessive ROS levels lead to OS which can reduce fertile capacity through lipid peroxidation of the membrane, sperm deoxyribonucleic acid fragmentation, and apoptosis

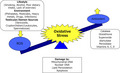

## 1 INTRODUCTION

**FIGURE 1:**
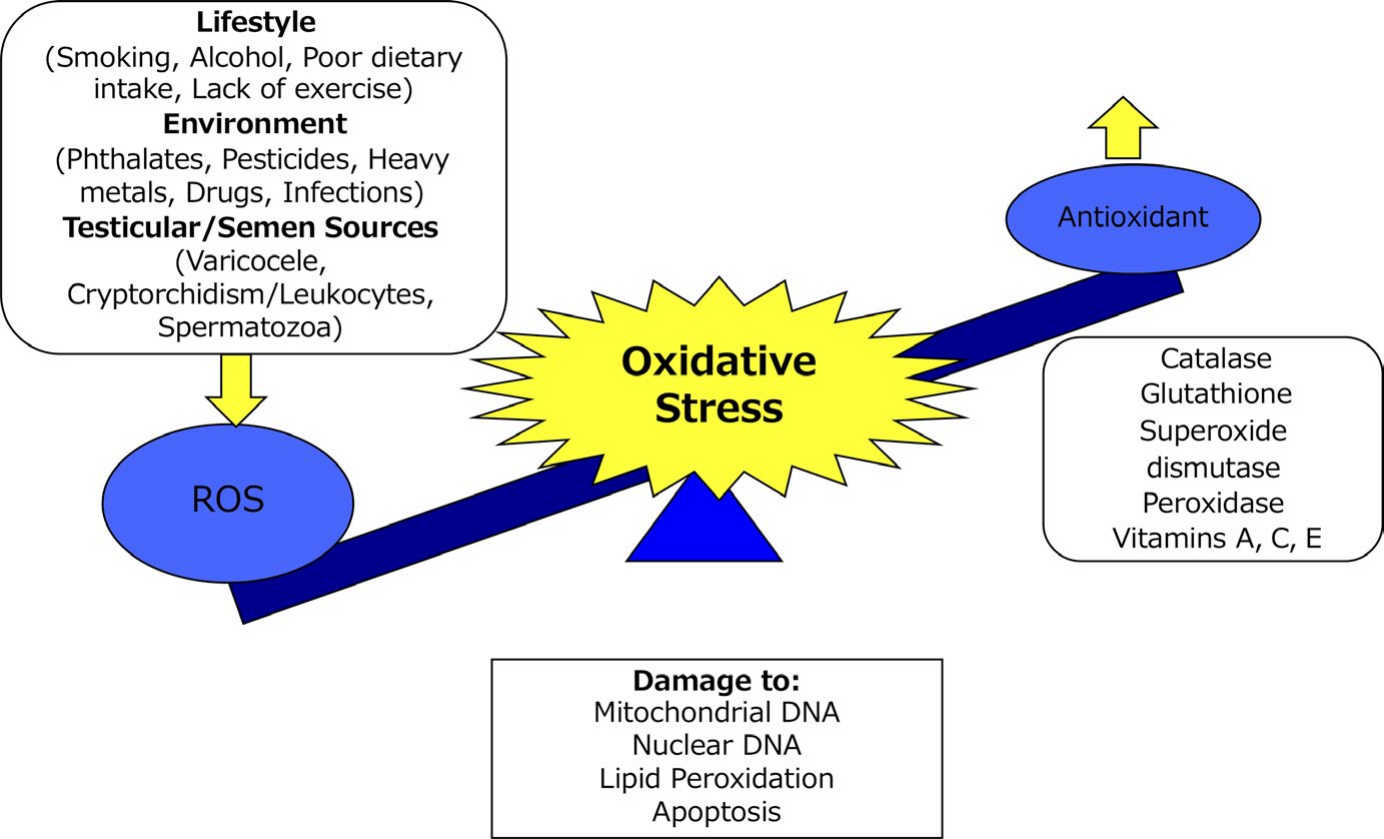
Factors that can cause oxidative stress. Oxidative stress results from a disturbance of homeostatic balance between ROS production and antioxidant capacity in seminal plasma

Infertility is defined by the International Committee for Monitoring Assisted Reproductive Technologies as the incapacity to conceive after at least one year of regular, unprotected, and well‐timed intercourse,^1^ and has become a global problem faced by one out of five couples. Although approximately 50% of these cases are associated with male infertility,^2^ the etiology of male infertility is multifactorial and the average incidence of unexplained male infertility is approximately 15%.^2^ Furthermore, approximately 40% of cases were unexplained in a recent survey on infertility among Japan males.^3^ Many studies have reportedly revealed the pathophysiologies of unexplained male infertility, but the causes of most cases remain unknown. Many studies on the effect of seminal oxidative stress (OS) on male fertile capacity have been reported since Aitken et al first reported reactive oxygen species (ROS) in washed human semen in 1987 using a chemiluminescence assay.^4^ OS results from a disturbance of homeostatic balance between ROS production and antioxidant capacity in seminal plasma in human semen ^5^ (Figure 1). It is well known that a small amount of ROS is vital for the steps involved in the essential physiological response of fertilization—sperm maturation, hyperactivation, capacitation, acrosome reaction of sperm, and sperm‐oocyte fusion.^6^, ^7^ However, lipid peroxidation (LPO) within the cellular membrane, deoxyribonucleic acid (DNA) fragmentation in nuclei and mitochondria, and apoptosis^8^ can occur when the production level of ROS gets excessive. All these events negatively affect sperm parameters,^9^, ^10^, ^11^, ^12^, ^13^, ^14^, ^15^ male fertility,^12^ and pregnancy outcome of their partners. Clinically, several studies have demonstrated that OS in human semen resulted in significantly poor assisted reproductive technology (ART) outcomes, such as lower fertilization rates, arrest of embryonic development, implantation failure, recurrent pregnancy loss, and lower live birth rates.^16^, ^17^, ^18^ Agarwal et al proposed the term and concept of “Male Oxidative Stress Infertility (MOSI)”,^19^ and it has been determined that there are many patients with MOSI among those who were previously classified as having idiopathic male infertility.^19^ Understanding the effects of ROS on male fertile capacity, measuring OS accurately, and treating patients based on their pathophysiologies will contribute to improving male infertility outcomes. The following short review will describe the pathophysiologies of OS and discuss its treatments.

## 2 PATHOPHYSIOLOGY OF ROS IN HUMAN SEMEN

ROS are typically classified as either free radicals (hydroxyl radicals (^.^OH), superoxide anion (^.^O_2_^−^), or peroxyl radicals (^.^RO_2_)), or non‐radicals (hydrogen peroxide (H_2_O_2_) or hypochlorous acid (HOCl)).^20^ Strictly speaking, reactive nitrogen species, such as nitric oxide (^.^NO), nitric dioxide (^.^NO_2_), and peroxynitrite (ONOO^−^), are considered a subclass of ROS. Free radicals are unstable short‐lived reactive chemical compounds that contain an unpaired valence electron. The electrons form pairs by depriving other compounds of an unpaired electron, which can cause oxidation. One of the representative free radicals, superoxide anion, reacts to form a precursor of hydroxyl radicals and hydrogen peroxide. Hydrogen peroxide is one of the non‐radical species and is not very reactive. However, it generates hydroxyl radicals,^21^ which are highly reactive and oxidative, when endogenous metal ions are present. It is therefore the most important factor causing oxidative damage, including DNA damage and in vivo cellular LPO, especially in human semen.

It has become apparent over the past 20 years that the physiological levels of ROS have an impact on several signaling pathways that regulate biological and physiological redox‐sensitive processes.^22^ These redox processes usually require ROS for interaction with the amino acid cysteine on proteins. ROS typically mediate cell proliferation and apoptotic pathways that regulate the cell cycle and programmed cell death. Likewise, ROS in semen plays a low‐level role as a second messenger in the fertilization processes, including sperm maturation, hyperactivation, capacitation, acrosome reaction, egg penetration, and sperm head decondensation.^6^, ^7^ Human sperm generates ROS through several pathways which induce cyclic adenosine monophosphate in sperm, activate tyrosine kinases, and increase the tyrosine phosphorylation level. The localization of tyrosine phosphorylation in the flagellum causes spermatozoal hyperactivation within the female genital tract; furthermore, it causes binding of the sperm to the zona pellucida which is essential for acrosome reaction.^6^, ^7^

OS caused by excessive ROS production has various detrimental effects on certain components of the human body, such as the cranial nervous system, cardiovascular system, digestive system, and endocrine and metabolic systems, even though low levels of ROS have important physiological functions.^23^, ^24^, ^25^, ^26^ An excess of seminal ROS has been reported in 30% to 80% of infertile men.^11^, ^15^, ^27^ It is well known that seminal OS induces LPO of the sperm cell membrane, sperm DNA fragmentation (SDF) and, consequently, apoptosis.

OS results from not only excessive ROS production but also from low antioxidant capacity. All human semen contains endogenously produced antioxidants in the seminal plasma to protect the sperm from OS via three mechanisms: prevention, interception, and repair. These antioxidants are classified as enzymatic and nonenzymatic antioxidants.^28^ Representative enzymatic antioxidants include superoxide dismutase (SOD), catalase, and glutathione peroxidase (GPX). Nonenzymatic antioxidants include ascorbic acid (vitamin C) and alpha‐tocopherol (vitamin E), coenzyme Q10, astaxanthin, myo‐inositol, urate, taurine, melatonin, transferrin, L‐carnitine, and lactoferrin.^28^, ^29^, ^30^ These antioxidants function as ROS scavengers to maintain redox homeostasis. The total antioxidant capacity (TAC) assay was often used to measure the total amount of nonenzymatic antioxidants in seminal plasma.^31^

## 3 MAIN SOURCES OF ROS

The two major sources of endogenous ROS in human semen are leukocytes (extrinsic ROS) in seminal fluid^4^, ^32^ and immature sperm (intrinsic ROS) having a morphologically abnormal head and cytoplasmic retention.^32^, ^33^, ^34^

When leukocyte chemotaxis and activation stimulate male genital tract inflammation and infection, extrinsic ROS is produced. ROS production occurs when leukocytes break down pathogens by activating the myeloperoxidase system.^27^ This excessive production of ROS by leukocytes can lead to OS in seminal fluid.

Intrinsic ROS is produced by abnormal and immature spermatozoa. Cytoplasm deposits in the mid‐piece fall off to cause cell elongation and condensation during the normal spermiogenesis process. The morphologically abnormal, immature spermatozoa retain the excess residual body containing large amounts of cytosolic glucose‐6‐phosphate dehydrogenase enzyme and produce intracellular nicotinamide adenine dinucleotide phosphate (NADPH). NADPH then produces ROS via NADPH oxidase called NOX5 located in the intramembrane.^35^ Therefore, immature spermatozoa are characterized by the presence of cytoplasm in the residual body of the mid‐piece which produces excessive ROS. It is very important to determining the source of excessive ROS production in semen because infiltrating leukocytes and immature spermatozoa have very different clinical implications.

Myeloperoxidase staining is effective for distinguishing granulocytes such as neutrophils, polymorphonuclear leukocytes from germ cells to determine the source of excessive ROS production in semen.^36^, ^37^ Leukocytes positive for peroxidase staining in semen stain brown, which reflects their capacity for producing excessive ROS through phagocytosis.^38^ These activated leukocytes increase NADPH production via the hexose monophosphate shunt, allowing them to produce 100 times more ROS than non‐activated leukocytes.^39^

On the other hand, nitroblue tetrazolium (NBT) staining is effective for detecting ROS production by immature spermatozoa.^40^ NBT is a yellow, water‐soluble, nitro‐substituted aromatic tetrazolium compound that reacts with cellular superoxide ions to form a blue‐colored formazan derivative, which can be observed either microscopically or spectrophotometrically using an enzyme‐linked immunosorbent assay (ELISA) plate reader. Oxidation within the cytoplasm helps transfer electrons from NADPH to NBT and reduces NBT to blue‐colored diformazan. The intensity of staining is therefore correlated with that of intracellular ROS.^41^ NBT staining is a user‐friendly technique used to predict the level of ROS production and detect the source of ROS production.

## 4 OTHER SOURCES OF ROS

Table 1 shows the well‐established external sources of ROS roughly classified into five factors. Many investigations have focused on the impact of these factors on seminal OS. For example, alcohol abuse reportedly causes excessive ROS production, partly due to undernutrition resulting in insufficient antioxidant intake.^42^ Alcohol abuse also decreases the success rate of IVF and increases the rate of miscarriage,^43^ and tobacco smokers could exhibit higher ROS production than nonsmokers.^44^ However, the mechanism of smoking toxicity is complicated because tobacco contains many kinds of chemical compounds, including nicotine, tar, carbon monoxide, and heavy metals.^45^ Many of these toxic compounds have an oxidizing effect and can induce in vivo chromosomal aberrations and SDF.^46^ Smoking can recruit proinflammatory leukocytes which increase seminal ROS levels. A small cohort study reported that smokers had 48% higher seminal leukocyte levels and 107% higher seminal ROS production than nonsmokers.^47^ The mean sperm DNA fragmentation index (DFI) of infertile smokers was also reported as being higher than in infertile nonsmokers (37.66% vs. 19.34%, *P* < .001).^48^ Furthermore, the levels of endogenous antioxidants, such as vitamins C and E, were decreased in the seminal plasma of smokers, which indicates lower protection against OS.^49^

**TABLE 1:** Origin of OS

Lifestyle
Smoking
Insufficient diet
Obesity
Alcohol
Age
Environmental
Pollution
Heavy metals
Heat
Phthalate
Mobile phone radiation
Infection
Genitourinary tract infection
Testicular
Clinical varicocele
Iatrogenic
Cryopreservation
Centrifugation
Drugs

Obesity is often associated with impaired spermatogenesis due to endocrinological abnormalities. Adipose fibroblasts contain aromatase which converts testosterone into estradiol. Moreover, cytokines are generated from adipose tissues leading to the recruitment of proinflammatory leukocytes and an increase in NADPH oxidase activity, which induces OS.^50^ Previous investigations have reported a positive correlation between body mass index and DFI.^51^

Environmental pollution and heavy metals can also induce OS. For example, phthalates are a class of chemicals used as plasticizers, which are also endocrine disrupters. They are widely found in polyvinyl chloride plastics and have been implicated in OS induction. Phthalate esters act as peroxisome proliferators and can produce H_2_O_2_ and other oxidants.^52^

Wearing tight‐fitting underwear, using a sauna, bathing for long periods of time, using a laptop on closed legs, and cycling^53^, ^54^, ^55^, ^56^ may result in an elevated scrotal temperature. The location of the scrotum keeps the temperature of the testes lower than body temperature by approximately 2°C.^57^ Increased scrotal temperature may inversely, but reversibly, affect spermatogenesis. Several authors have reported a correlation between scrotal heat stress and SDF. Furthermore, intermittent heat exposure reportedly impedes spermatogenesis more than continuous heat exposure.^56^ Immature spermatozoa may produce excessive ROS since the process of spermiogenesis is susceptible to heat stress and heat stress may alter the normal function of the epididymis.^58^ OS may contribute to spermatogenesis suppression in response to heat.

Previous investigations have shown the effect of mobile phone radiation on increasing ROS production and decreasing the activity of antioxidants, such as catalase, SOD, and glutathione peroxidase (GPX).^59^Recently, Gautam et al demonstrated that significant increase in ROS and lipid peroxidation level with simultaneously decrease in sperm count, alterations in sperm tail morphology were observed in the male Wistar rats which were exposed to 3G mobile phone for 45 days.^60^

Approximately 15% of the general male population—30%–40% of men with primary infertility, and up to 80% of men with secondary infertility—are diagnosed as having clinical varicocele.^61^ Recent meta‐analyses have shown that varicocele repair significantly improves seminal parameters^62^, ^63^ and is considered as one of the most common causes of surgically treatable male infertility.^64^ OS is one of the major contributors to male infertility in men with varicocele, and many investigations have demonstrated elevated levels of SDF in men with varicocele.^65^ One of the main mechanism is considered to be a protamination and chromatin compaction disorder during the spermiogenesis process, which elevates the sensitivity of affected cells to OS causing defective spermatogenesis and SDF.^66^ Most studies reported that the ROS production level of semen in men with varicocele was increased compared to the controls.^67^ Furthermore, the endogenous antioxidant level in seminal plasma was decreased in patients with varicocele.^68^

## 5 IMPACT OF OS ON SPERMATOZOA

A disturbance of the homeostatic balance between ROS and antioxidants occurs when highly reactive ROS exceed the antioxidant defense systems, and it can lead to the development of OS. This can have detrimental effects on sperm, such as LPO, SDF, and apoptosis. We describe each effect below.

### 5.1 Lipid peroxidation

Sperm cells have abundant lipids in their plasma membrane mostly in the form of polyunsaturated fatty acids (PUFAs), especially docosahexaenoic acid, where six double bonds between their methylene groups are not conjugated. Increased ROS production induces PUFA peroxidation within the sperm cell membrane,^69^ which induces cell dysfunction due to loss of membrane fluidity and integrity required for successful sperm‐oocyte fusion after the capacitation and acrosome reaction biochemical cascades.^70^ Byproducts of LPO bind to and disrupt mitochondrial proteins of the electron transport chain apart from disrupting the sperm cell membrane, which leads to electron leakage and consequently decreases mitochondrial membrane potential, decreases ATP production, and decreases sperm motility.^71^ LPO has three phases: the first phase is “initiation,” which is the extraction of hydrogen atoms from the carbon‐carbon double bonds of an unsaturated fatty acid to propel free radicals. The second phase is “propagation,” which is the formation of lipid radicals followed by their rapid reaction with oxygen to form peroxyl radicals.^72^ When metals such as copper and iron are present, the peroxyl radicals can again abstract a hydrogen atom from an unsaturated fatty acid to produce a lipid radical and lipid hydrogen peroxide.^73^ The last phase is “termination,” where these formed radicals react with successive lipids and generate cytotoxic aldehydes and other end products.

The main products of LPO are 4‐hydroxynonenal (4‐HNE), malondialdehyde (MDA), and acrolein. One method used to quantify LPO is MDA measurement according to the spectrophotometric thiobarbituric acid reaction (TBAR) test. MDA is an essential biomarker for analyzing and monitoring PUFA peroxidation levels.^69^, ^74^

### 5.2 Sperm DNA fragmentation

Excessive ROS production and decreased antioxidant levels in semen can also lead to SDF. OS can damage sperm DNA directly or indirectly through sperm caspase and endonuclease activation. SDF is caused by DNA vulnerability due to a chromatin compaction error during the spermiogenesis process, which causes a substitution failure of chromatin structure from histone to protamine. This damage is due to ROS exposure after spermiation, during comigration of spermatozoa from the seminiferous tubules through the rete testis to the cauda epididymis. This results in the formation of 8‐OH‐guanine and 8‐OH‐2'‐deoxyguanosine (8‐OHdG),^75^ which is an oxidized guanine adduct. Increased 8‐OHdG concentration correlates significantly with DNA fragmentation and strand breaks.

DNA has a double‐helix structure, and DNA fragmentation can occur in both the single‐stranded (ss‐) and double‐stranded (ds‐) forms. DNA repair can only occur during specific stages of spermiogenesis, and the repair mechanisms are no longer activated during nuclear condensation in the epididymis. The next opportunity for ss‐DNA break repair is by the human oocyte, which is a critical step in embryo development, although the ability to repair SDF decreases with advanced maternal age.^76^ The ds‐DNA break results in genomic instability and apoptosis in the absence of repair.^77^ The presence of unrepaired SDF above the critical threshold reportedly has a detrimental effect on embryo development and pregnancy outcome—also called the “late paternal effect”.^78^ In a cleavage‐stage embryo, major activation of embryonic genome expression begins on the second day of human embryo development (the 4‐cell stage), and embryogenesis switches from maternal factor dependence to the embryo's own genome dependence.^79^ Therefore, a spermatozoon with SDF negatively affects blastulation, implantation, and pregnancy outcomes after fertilization. Furthermore, Kuroda et al reported that OS also had an adverse effect on cleavage embryo development, called “early paternal effect”.^80^ Several investigations have reported the relationship between ART and SDF outcomes. A previous meta‐analysis demonstrated that SDF was inversely correlated with pregnancy outcome [relative risk (RR): 0.81; 95% confidence interval (CI): 0.70‐0.95; *P* = .008] and positively correlated with miscarriage (RR: 2.28; 95% CI: 1.55‐3.35; *P* < .0001).^81^ Proper measurement and management can reduce the burden on couples since SDF can cause recurrent pregnancy loss.

### 5.3 Apoptosis

Apoptosis via multiple cell death signaling and regulatory pathways is known as physiologically programmed cell death due to DNA fragmentation. ROS‐induced ds‐DNA breaks can result in apoptosis. ROS also disrupts the mitochondrial membranes so that they release signaling molecule cytochrome C, which can activate the apoptotic caspases and annexin‐V binding to phosphatidylserine. High cytochrome c levels in seminal plasma may suggest significant damage to mitochondria caused by high levels of ROS in infertile patients.

## 6 EVALUATION OF OS IN HUMAN SEMEN

As previously reported in the literature,^19^ OS has been linked to unexplained and idiopathic male infertility, and measurement of OS is essential for its subsequent management and treatment. Currently, more than 30 different assays have been described to measure seminal OS. They can be classified as direct or indirect assays (Table 2). Direct assays quantify the levels of ROS directly. Indirect assays quantify the adverse effects of OS, such as SDF or LPO levels.^82^

**TABLE 2:** Direct and indirect semen assays of ROS

Direct assays	Indirect assays
Chemiluminescence	Myeloperoxidase (Endz) test
Nitroblue tetrazolium test	8‐OHdG
Oxidation‐reduction potential	Thiobarbituric acid reaction (TBAR) test
Flow cytometry	Total antioxidant capacity (TAC) assay

### 6.1 Direct measurement assays for OS

**FIGURE 2:**
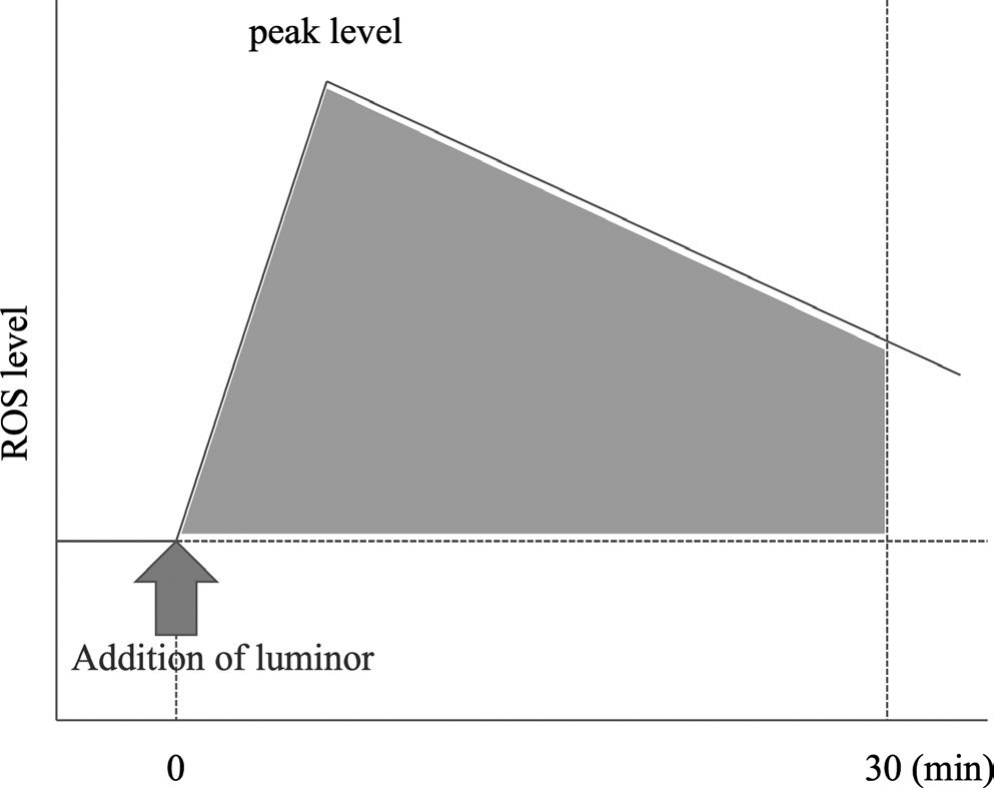
Measurement of ROS by chemiluminescence method. ROS production levels were calculated as the integrated chemiluminescence for 30 min after the addition of luminol (5‐amino‐2,3‐dihydro‐1,4‐phtalazinedione) to unwashed semen after baseline subtraction

The chemiluminescence method is one of the direct assays used to quantify seminal ROS levels. Takeshima et al^11^ and Yumura et al^15^ calculated chemiluminescence using a luminometer, specifically Luminometer 1251™ (LKB Wallac, Turku, Finland) and the Monolight 3010™ Luminometer (BD Biosciences Pharmingen, Ltd.) respectively, after adding 40 μL of 100 mmol/L luminol (5‐amino‐2,3dihydro 1,4‐phtalazinedione) to 500 μL of unprocessed semen. The levels of ROS production were calculated automatically by subtracting the area under the baseline from the total integrated chemiluminescence for 30 minutes after adding luminol to unprocessed semen, and expressed as relative light units (RLU)/200 s/10^6^ of spermatozoa (Figure 2).^11^, ^15^ There are a variety of luminometers available, which include single and double tube luminometers, and multiple tube luminometers, each with its own strengths and weaknesses, which can be used according to the number of samples processed.^83^

As described above, the NBT assay is also a cost‐effective, user‐friendly, and sensitive direct assay. Unlike chemiluminescent assays, the NBT assay can assess seminal ROS levels and microscopically or spectrophotometrically determine the source (immature sperm or leukocytes) of ROS. Diformazan crystal concentration has a positive correlation with intracellular ROS level, and the location of the crystals reveals the cellular source of ROS in semen.^40^, ^41^

### 6.2 Indirect measurement assays for OS

MDA is one of the byproducts of LPO, and it can be commonly measured as an indicator of the LPO level. Sperm MDA concentration is measured with a TBAR assay through spectrophotometry or fluorometry.^84^ Sperm MDA levels have a positive correlation with ROS production levels in the semen of infertile men^84^; however, this assay is a nonspecific test providing only post hoc measurement of LPO.

8‐OHdG is a product of oxidative DNA damage that is cleaved by the specific enzyme after 8‐hydroxylation of the guanine base; it is also used as a sensitive biomarker for ROS‐induced oxidative DNA damage in human sperm.^85^ 8‐OHdG can be measured by ELISA in seminal plasma and can be quantified by immunohistochemical staining in testicular tissue.^86^

To quantify the total amount of antioxidants, TAC assay based on the ability of antioxidants in the seminal plasma samples to inhibit 2,2'‐azino‐di‐ [3‐ethylbenzhiazoline sulfonate] (ABTS) oxidation to ABTS^+^ was often used. This is because measuring each specific antioxidant provided limited information on assessed TAC.^87^, ^88^

Seminal TAC can be measured as the sum of antioxidant activities available in seminal plasma using enhanced chemiluminescence or colorimetric techniques.^88^ Previous studies reported that a low level of seminal TAC was associated with male infertility.^89^ Mahfouz et al reported that infertile patients had lower seminal plasma TAC levels compared to the proven fertile and donor group.^90^ However, the ROS‐TAC score which calculates the balance between oxidation and reduction potential^82^ is superior for measuring ROS and TAC alone and is more suitable for predicting MOSI.^91^ The ROS‐TAC score is a parameter formulated using the ratio of standardized ROS production level in washed sperm suspensions and standardized TAC in seminal plasma using a principal component analysis. A cutoff value of 30 was determined as the lower end of a normal range of ROS‐TAC score, and patients with lower scores are assumed to be at risk for infertility.^92^ However, TAC assays cannot be performed routinely, as they require expensive equipment and advanced technical skills while only measuring nonenzymatic antioxidants.^13^ MiOXSYS^®^ has the potential to substitute TAC assay, as it can measure simultaneously oxidation‐reduction potential (ORP) more easily and in a shorter time.

### 6.3 Novel measurement assays for oxidation‐reduction potential

In contrast to the methods described above, ORP measurement is a direct assay for quantifying OS with the Male Infertility Oxidative System (MiOXSYS®) analyzer (Aytu BioScience Inc, California, USA), which is a novel, user‐friendly, and less expensive technology for evaluating the balance between the oxidative and reductive capacities of human semen.^93^, ^94^ Therefore, studies on ORP measurements have been increasingly reported^93^, ^94^ in recent years, and static ORP (sORP) values have been calculated and expressed as mV/10^6^ spermatozoa/mL. A higher sORP level indicates an imbalance of increased ROS production compared to all antioxidants available in semen, thus indicating the presence of OS. Agarwal et al set the cutoff level at 1.34 mv/10^6^ spermatozoa/mL for identifying normal/abnormal semen quality.^95^ This MiOXSYS^®^ system is therefore a very useful diagnostic tool for screening oxidative male infertility.

## 7 MANAGEMENT AND PREVENTION FOR MOSI

There are many types of precautionary measures, such as those related to lifestyle and environmental modifications and treatment options (eg, sperm selection techniques) that can be used to minimize the adverse effects of OS on the reproductive function.

### 7.1 Sperm selection techniques

Sperm selection techniques that manage OS by removing sperm with oxidative DNA damage include density gradient centrifugation (DGC),^96^ electrophoretic separation,^97^ intracytoplasmic morphologically selected sperm injection,^98^ hyaluronic acid binding assay,^99^ and annexin‐V magnetic‐activated cell separation.^100^ Of these, DGC is a commonly used technique that can separate mature spermatozoa from immature spermatozoa, leukocytes, bacteria, and cell debris, which are origins of toxic ROS as a sperm preparation method for ART. Takeshima et al reported that DGC remove ROS, supporting that this method can select motile spermatozoa without enhancing OS.^96^

### 7.2 Lifestyle and environmental modification

The lifestyle and environmental factors that increase ROS production are shown in **Table** **1**. Patients may quit smoking,^101^ avoid alcohol abuse and lose weight through balanced diet and moderate exercise,^102^ and decrease their exposure to phthalates^52^ in order to minimize extrinsic ROS production. It is also well‐established that increased scrotal temperature^55^, ^56^ and exposure to harmful substances^103^ leads to an increase in OS and can have detrimental effects on fertile capacity. Among them, activities raising the temperature of the scrotum should be considered. Avoiding wearing tight‐fitting underwear, bathing in hot water for a long period, using saunas, using a laptop on closed legs, and cycling can minimize OS.^53^, ^54^, ^55^, ^56^ Furthermore, adequate aeration and use of protective equipment in the workplace to reduce exposure to noxious chemicals and vapors that induce OS are also an effective measure to minimize OS. Mobile phone electromagnetic waves were reported to increase ROS production and decrease antioxidant activities^59^; therefore, storing it somewhere other than in a trouser pocket may minimize OS.^104^

### 7.3 Shorter interval of ejaculatory abstinence

Because SDF occurs after spermiation during transfer of spermatozoa from the seminiferous tubules through rete testis to the cauda epididymis due to the ROS exposure, they can be affected by a harmful seminal microenvironment of OS while stored in the epididymis and after ejaculation. Therefore, increasing ejaculation frequency and decreasing the storage interval of spermatozoa in the epididymis may reduce sperm exposure to toxic ROS, thereby increasing sperm motility and decreasing SDF. Several studies have shown that a shorter interval between ejaculatory abstinence contributed to a lower seminal ROS and sperm DFI.^105^, ^106^ A shorter interval between ejaculatory abstinence may improve sperm quality and DNA integrity by reducing sperm exposure to excessive ROS in the epididymis.

### 7.4 Oral antioxidant therapy

Oral antioxidant therapies such as lactoferrin and transferrin were found to inhibit the formation of ROS and scavenging antioxidants such as vitamins C and E eliminate ROS and improve sperm parameters and pregnancy outcomes in patients with OS and SDF. According to a systematic review by Gharagozloo and Aitken, 20 studies highlighting the effects of antioxidant supplementation on measures of OS in semen were reviewed, and significant reduction in OS or SDF and improvement in sperm motility, particularly in asthenospermic men after antioxidant treatment, was observed in 19 out of 20 studies.^107^ Moreover, there have been systematic reviews on antioxidant therapy.^108^ According to the Cochrane database, 61 randomized controlled trials comparing the effect of antioxidants and a placebo in a population of 6,264 infertile men were reviewed. The results demonstrated that antioxidants could increase clinical pregnancy and live birth rates. Typical oral antioxidant therapies are vitamin C alone (400‐1000 mg/day),^109^ vitamin E alone (300‐600 mg/day),^110^, ^111^or a combination of vitamin C and E.^112^, ^113^ Vitamin C and E act synergistically, and several studies have reported the advantageous effect of complex antioxidants on reducing SDF and increasing clinical pregnancy rates.^112^, ^113^ Zinc is an essential element for spermatogenesis and sperm DNA synthesis. It also prevents LPO and acts as a component of SOD.^114^, ^115^ Selenium is also an essential component of the GPX selenoproteins.^116^ Several studies reported that antioxidants combined with zinc and selenium may lower SDF and increase clinical pregnancy rates.^117^ L‐Carnitine and coenzyme Q10 are powerful antioxidants that prevent LPO and SDF. Meta‐analysis and other studies showed that both compounds improved conventional sperm parameters.^118^, ^119^, ^120^, ^121^, ^122^ Several studies on supplementation with a combination of antioxidants have also been reported.^123^, ^124^, ^125^ These antioxidant therapies and outcomes were shown in Table 3. While there have been many studies on the favorable outcomes of these treatments, there have been also several studies with negative outcomes and possible toxic effect when overused.^126^, ^127^ Therefore, the efficacy is still controversial. True efficacy of these treatments should be validated by large‐scale nonrandomized two‐arm studies between OS‐positive and ‐negative groups.

**TABLE 3:** Various antioxidant therapies and outcomes

Antioxidants	Outcomes	Reference
Vitamin C	low vitamin C intake: DFI increased high vitamin C intake: DFI decreased	108
Vitamin E	LPO decreased sperm motility increased	109
	zona binding rate increased	110
Vitamin C + Vitamin E	DFI decreased	111
	DFI decreased	112
L‐Carnitine	sperm density, motility increased DFI decreased	119
Coenzyme Q10	sperm density, motility, TAC increased ROS level, DFI decreased	120
Vitamin C + Vitamin E + Coenzyme Q10	sperm density, motility increased	122
Vitamin C + Vitamin E + Zinc +Selenium + L‐Carnitine + Coenzyme Q10 + N‐acetyl L‐cysteine and other components	sperm density, motility increased DFI, ORP decreased	123
Vitamin C + Vitamin E + Zinc +Coenzyme Q10 + L‐Carnitine + Astaxanthin	total motile sperm count increased sperm density, motility no change	124

### 7.5 Varicocele repair

As described earlier, much evidence suggests that OS and increasing SDF are the major contributors to infertility in men with a varicocele.^66^ In addition, much evidence also suggests that varicocelectomy in men with clinically palpable varicocele and infertility significantly improves male fertile.^64^, ^128^ There are several surgical options for repairing varicocele, but microsurgical varicocelectomy by the subinguinal approach is considered the gold‐standard technique for varicocele repair because it has the lowest incidence of postoperative recurrence and complications when compared to other procedures.^129^ Many studies have shown that varicocelectomy lowers seminal OS and ameliorates SDF. In addition, varicocelectomy has been reported to significantly increase antioxidant levels indirectly.^130^

### 7.6 Testicular sperm extraction

As mentioned above, spermatozoa in semen are affected by ROS during the ejaculation process. The testis is protected by substantial antioxidant systems, but spermatozoa are released from the Sertoli cells during the spermiation process and migrate from the seminiferous tubules through the rete testis to the epididymis; they become susceptible to OS.^131^ The SDF level in testicular sperm has been reported to be one third lower than in ejaculated sperm.^132^ Testicular sperm extraction (TESE) is a procedure by which sperm is surgically retrieved from the testis of patients with azoospermia, cryptozoospermia, or ejaculatory disorders. ICSI using testicular sperm has a higher implantation and clinical pregnancy rates than ICSI using ejaculated sperm.^133^, ^134^ However, testicular sperm has a significantly higher aneuploidy rate than ejaculated sperm.^132^ Therefore, this method should be performed with limited indication of recurrent ART failure and severe oligozoospermia cases.

## 8 CONCLUSIONS AND FUTURE DIRECTIONS

Male patients with idiopathic/unexplained infertility should be screened for MOSI as mentioned above using an efficient, inexpensive, and high sensitivity/high‐specificity ORP assay as a screening test.^19^, ^94^ Those who are MOSI‐positive should then undergo a more extensive examination to identify the treatable factors.

In recent years, advances in proteomics technology have led to the discovery of many protein biomarkers of disease to elucidate the pathological condition and establish treatment methods.^135^, ^136^ Protein identification in spermatozoa and seminal plasma in semen samples exposed to OS will help understanding the biological pathways associated with male infertility and may lead to the discovery of new biomarkers of idiopathic male infertility. HSPA2, a member of HSP70 family, as a heat shock protein, is reportedly a key protein underexpressed in ROS‐positive sperm. HSPA2 is a protein located in the sperm tail and is involved in spermatogenesis.^137^ Underexpression of DJ‐1 (which removes ROS such as hydrogen peroxide and inhibits apoptosis) has been determined through seminal plasma proteome analysis in ROS‐positive semen.^135^ These proteins could be possible biomarkers for OS in semen. Environmental factors, such as endocrine disruptors such as BPA (bisphenol A), DBP (dibutyl phthalate), and DEHP (bis (2‐ethylhexyl phthalate)) in plastics, can generate OS and cause epigenetic DNA methylation.^138^ Moreover, oxidative damage can cause epigenetic changes through a variety of mechanisms, including DNA methylation, histone modifications, and chromatin remodeling. It has been reported that DNA methylation deficiency caused by deficiency of methyltransferase KMT2D is impaired by ROS and is implicated in spermatogenesis, Sertoli cell only syndrome, and the incidence of testicular cancer.^139^ Exploring biomarkers and epigenetic changes may lead to additional treatment and screening options in the future.

## DISCLOSURES

*Conflicts of interest*: The authors report no declarations of interest. Human/animal rights statements and informed consent: This article does not contain any studies with human and animal subjects performed by any of the authors.

*Approval by Ethics Committee*: This research was supported by the Ethics Committee of Yokohama City University Medical Center.
